# An Evaluation of Holiday Weight Gain Among Elementary-aged Children

**DOI:** 10.4021/jocmr414w

**Published:** 2010-08-18

**Authors:** Paul Branscum, Gail Kaye, Paul Succop, Manoj Sharma

**Affiliations:** a The University of Cincinnati, Health Promotion and Education, Cincinnati, OH 45221-0068, USA; b The Ohio State University, Department of Human Nutrition, Columbus, OH 43210, USA; c The University of Cincinnati, Department of Environmental Health, Cincinnati, OH 45221-0068, USA

## Abstract

**Background:**

Previous studies suggest adults gain extra weight during the holiday season, however, few studies have been done with children during this time. The purpose of this study was to evaluate gains in growth among elementary children, and compare differences by gender and weight status.

**Methods:**

Children’s (n = 90) height and weight were measured before and after their holiday break. Height, weight and body mass index (BMI) and body mass index-percentiles (BMI-%) were evaluated and compared by groups using repeated measures ANCOVA’s.

**Results:**

On average, children grew 0.82 cm (0.32 in), and gained 0.56 kg (1.2 lbs) and 0.28 BMI units, however the average BMI-% slightly decreased by 0.4%. Overweight and obese children gained significantly more weight, BMI units and BMI-% units compared with normal weight children.

**Conclusions:**

This study supports that the holiday period may be an important time to target children, especially those who are already overweight and obese.

**Keywords:**

Holiday weight gain; Childhood obesity

## Introduction

The prevalence of childhood overweight and obesity continues to increase among youth [1]. In efforts to reduce childhood obesity, many health-promoting interventions targeting modifiable risk factors, such as diet and physical activity, have been employed but have shown varying degrees of success [2, 3]. While it is important to modify such risk factors, there may be environmental risk factors, such as seasonal periods, that are also important. Currently, the context of how seasonal periods affect weight status among children is not well understood. Some point to the in-school environment as an especially obesogenic time. With in-school cafeterias and vending machines, children have access to many competitive foods that are not required to meet USDA nutrition standards that federally subsidized meals are required. Children are also given little time to engage in physical activities as P.E. classes and recess are being cut [4, 5]. Others point to the out of school environment as an at risk time for weight gain. At home, children engage in a large amount of screen time [6], frequent convenient stores and fast food restaurants that serve energy-dense/nutrient poor foods [7], and are monitored less by parents as the need increases for both parents or guardians to have full-time employment [4].

To determine if the in or out of school time contributed more to childhood overweight, Von Hipple and colleagues compared gains in body mass index (BMI) among kindergarten and 1st grade children during the school and summer vacation months [4]. During the school year, kindergarten and 1st grade children’s BMI increased by 0.02 units and 0.033 units per month respectively, while during the summer vacation months the average increase more than doubled, with an average increase of 0.076 units per month [4]. Another study with middle and upper elementary American Indian children found somewhat similar results. Investigators reported that over the summer vacation, 3rd and 5th grade girls BMI and weight significantly increased, and 4th grade boys BMI significantly increased. Overweight and obese children also experienced a significantly higher gain in their BMI compared with their normal weight peers [8]. Together, these studies suggest that children are at greater risk for gaining extra weight during unstructured, out of school months.

Another unstructured-out-of-school time, albeit shorter in length compared with summer recess, is the holiday break, which typically spans from early December to early January. Adults commonly believe they gain 5 pounds during these months, however studies have shown actual weight gain is typically much less [8]. This time may be especially important to monitor children’s weight due to changes in eating and physical activity patterns that often accompany the holidays. It is generally agreed that during this time more holiday foods are eaten, which tend to be calorically dense. Bad weather conditions can also encourage children and families to stay in-doors and engage in more sedentary activities. In the study previously mentioned, Smith and colleagues evaluates changes in BMI during this time, and found that a small but significant increase in BMI was observed among fifth grade children [8]. It is important to note however, that this study used a homogenous group of American Indian children, making it difficult to generalize to children belonging to other racial groups.

While previous studies indicated a trend towards higher weight gains during the unstructured-out-of-school month, no studies reported children’s BMI-percentile (BMI-%) as an indicator of weight status. Evaluating ‘excess’ or ‘extra’ weight gain can be difficult among children since they are naturally growing, and absolute BMI does not account for normal growth as well as BMI-% [9]. The purpose of this study was to evaluate gains in height, weight, and BMI during the holidays, and compare these results with childrens BMI-% to control for normal growth. Since previous research found differences among genders and normal and overweight children, differences between these groups were also explored.

## Materials and Methods

### Sample

This sample consisted of 91 3rd, 4th and 5th grade Midwestern public elementary school children. To participate, children were required to have written parental consent and give verbal assent. Height and weight were measured twice by a team of eleven trained dietetic interns attending a large Midwestern University. The first measurements were taken in early December before childrens holiday break, and the second measurements were taken in early/mid January after children returned to school.

### Instruments and measures

Height and weight were measured without shoes and minimal clothing (for exemple, no sweatshirts or coats) to facilitate correct positioning of the body. Height was measured with a Seca 214 portable stadiometer to the nearest 0.1 cm. Body weight was measured on an electronic digital scale (Tanita HD 317) to the nearest 0.1 kg. BMI-% was calculated by entering childrens height, weight, date of birth and date of measurement into the CDC BMI-% calculator for children and teens (http://apps.nccd.cdc.gov/dnpabmi/Calculator.aspx) for both time points.

### Data analysis

To evaluate changes in height, weight, BMI and BMI-% over time, 4 separate one-way repeated measures ANCOVA were conducted. Gender and weight status were included as possible interaction effects. For weight status, children were stratified by ‘normal’ and ‘overweight/obese children’ with normal weight children being those with a BMI-% between 5th and 85th and overweight/obese children being those with a BMI-% greater than the 85th. An alpha (α) of 0.05 was set to determine statistical significance. Partial eta squared (η^2^) was also reported to determine strength of association with an η^2^ of 0.01 indicating a small effect, 0.06 indicating a medium effect, and 0.14 indicating a large effect. All data (σ) were analyzed using PASW software (PASW version 18.0, SPSS Inc., Chicago, IL, 2008). These research methods were approved by the Ohio State University Institutional Review Board on August 8th, 2008.

## Results

Overall, there were more boys (54%) than girls (46%), and on average children were 9.18 years old (σ = 0.93). Race and ethnicities of the children included Caucasian (45%), African American (39%), Hispanic (6%) and all remaining children were classified as other (10%). Compared with the national average, the prevalence of overweight and obesity was higher in this sample with 48% either overweight or obese.

On average, there were 37 days (or a little over 5 weeks) between pre and post holiday measurements. During this time children gained 0.56 kg (1.2 pounds), grew 0.82 cm (0.32 inches), gained 0.28 units of BMI, however the average BMI-% slightly decreased by 0.4% (Table 1). One-way ANCOVAs showed a significant main effect for all variables but BMI-%: (a) height (F[1,84] = 47.78, P = 0.007); (b) weight (F[1,84] = 59.28, P < 0.001); (c) BMI (F[1,84] = 59.28, P < 0.001); and (d) BMI-% (F[1,84] = 16.89, P > 0.05). For all four variables there were no significant differences between genders, however there were significant interaction effects for all variables but height, by weight status, with overweight children gaining more weight, BMI units and BMI-% units than normal weight children (Table 2).

**Table 1:** Gains in Height, Weight, BMI and BMI-% Among Children Stratified by Gender and Weight Status

		Pre-Holiday M (SD)	Post Holiday M (SD)	Difference
Height (cm)	Overall	139.12 (8.5)	139.88 (8.5)	+0.76
	Female	141.75 (8.5)	142.65 (8.4)	+0.90
	Male	136.82 (7.9)	137.47 (7.9)	+0.65
	Normal Weight	137.09 (8.7)	137.97 (8.7)	+0.88
	Overweight/Obese	141.23 (7.8)	141.88 (7.9)	+0.65
Weight (kg)	Overall	40.64 (12.9)	41.20 (13.2)	+0.56
	Female	43.61 (13.9)	44.24 (14.1)	+0.63
	Male	38.04 (11.4)	38.55 (11.9)	+0.51
	Normal Weight	32.23 (6.1)	32.58 (6.2)	+0.30
	Overweight/Obese	49.43 (12.2)	50.21 (12.6)	+0.78
Body Mass Index	Overall	20.48 (5.2)	20.76 (5.3)	+0.28
	Female	21.17 (5.7)	21.49 (5.7)	+0.32
	Male	19.88 (4.7)	20.14 (4.9)	+0.26
	Normal Weight	16.79 (1.6)	16.97 (1.6)	+0.18
	Overweight/Obese	24.36 (4.8)	24.73 (5.0)	+0.37
Body Mass Index Percentile	Overall	73.91 (26.0)	73.26 (27.1)	-0.65
	Female	76.27 (25.5)	75.73 (26.5)	-0.54
	Male	71.85 (26.5)	71.10 (27.7)	-0.75
	Normal Weight	53.99 (22.1)	52.59 (23.2)	-1.40
	Overweight/Obese	94.74 (4.1)	94.88 (4.3)	+0.14

Overall (n = 88); Female (n = 41); Male (n = 47); Normal Weight (n = 45); Overweight/Obese (n = 43)				

**Table 2:** Type III Sum of Squares and Effect Size for Changes in Height, Weight, BMI and BMI-%

	Type III Sum of Squares	df	F	P-value	η^2^
Height (Main Effect)	26.27	1	47.8	0.007	0.84
Height * Sex	0.71	1	1.3	ns	
Height * Wight Status	0.63	1	1.15	ns	
Height * Sex * Weight Status	0.15	1	0.27	ns	
Error	46.19	84			
					
Weight (Main Effect)	14.43	1	59.36	< 0.001	0.414
Weight * Sex	0.13	1	0.54	ns	
Wight * Weight Status	1.86	1	7.66	0.007	0.084
Weight* Sex * Weight Status	0.13	1	0.52	ns	
Error	20.43	84			
					
BMI (Main Effect)	3.47	1	59.28	< 0.001	0.414
BMI * Sex	0.024	1	0.40	ns	
BMI * Weight Status	0.37	1	6.47	0.013	0.072
BMI * Sex * Weight Status	0.34	1	0.58	ns	
Error	4.92	84			
					
BMI-% (Main Effect)	16.89	1	2.9	ns	
BMI-% * Sex	0.21	1	0.036	ns	
BMI-% * Weight Status	25.3	1	4.3	0.040	0.049
BMI-% * Sex * Weight Status	0.55	1	0.095	ns	
Error	487.97	84			

## Discussion

These findings suggest that growth among children during the holiday season can be mostly attributed to normal growth, except among overweight and obese children. It is a common belief among adults that they gain extra weight during the holiday season. While little research has been done evaluating this belief, one study found on average adults gained 0.37 kg (0.81 pounds) during the 6-week period from thanksgiving to news years [10]. If adults indeed gain extra weight during this time, then it is probable to assume their children would concurrently be at the same risk. In this study we found that on average children’s height, weight and BMI significantly increases during the holidays, but their BMI-% did not. This indicates that children overall do not experience excess weight during the holidays. However, a significant interaction effect by weight status for weight, BMI and BMI-% but not height, suggests overweight and obese children gain more weight during this time than their normal weight peers. The strength of association was also medium for all variables, indicating these gains are clinically relevant.

This study is not without limitations. While using BMI-% which has been reported as an optimal indicator for body adiposity among children [9], percentiles are bounded between the 1% and 99%. Six children (n = 6) had a BMI-% at 99% in this study, which makes it difficult to capture their variability. Children in this study also lived in the suburbs of a Midwestern city. Since environmental cues can strongly influence children’s access to outdoor activities, children living in warmer climates may not experience this problem. As noted in a previous study, measuring seasonal variations in weight gains can be difficult because while children are no longer in their structured school environment, it is difficult to know what environment children were exposed to [4]. It is likely that some children had very little structure and others could have been taken to day care centers that provide a similar structure as school.

Developing intervention strategies to prevent and treat childhood overweight and obesity is an area ripe for research. Furthermore, identifying seasonal periods and times when children are at greater risk for weight gain can help future researchers, dietitians and practitioners target and tailor intervention messages. This study supports that the holiday period may be an important time to target children, especially those who are already overweight and obese. Future research in this area is greatly needed to determine if this phenomenon is limited to certain geographical regions, and how much of this weight gain is mediated by diet and physical activity.
